# Consequences of Grazing Cessation for Soil Environment and Vegetation in a Subalpine Grassland Ecosystem

**DOI:** 10.3390/plants11162121

**Published:** 2022-08-15

**Authors:** Olga Gavrichkova, Gaia Pretto, Enrico Brugnoli, Tommaso Chiti, Kristina V. Ivashchenko, Michele Mattioni, Maria Cristina Moscatelli, Andrea Scartazza, Carlo Calfapietra

**Affiliations:** 1 Research Institute on Terrestrial Ecosystems, National Research Council, 05010 Porano, Italy; 2 Department for Innovation in Biological, Agrofood and Forest Systems, University of Tuscia, 01100 Viterbo, Italy; 3 Institute of Physicochemical and Biological Problems in Soil Science, 142290 Pushchino, Russia; 4 Department of Landscape Design and Sustainable Ecosystems, Agrarian-Technological Institute, Peoples’ Friendship University of Russia (RUDN University), 117198 Moscow, Russia; 5 Research Institute on Terrestrial Ecosystems, National Research Council, 56124 Pisa, Italy

**Keywords:** pasture, C cycle, C sequestration, management, dungs, livestock, biodiversity, functioning

## Abstract

Areas covered by seminatural grasslands have been in constant decline for decades in Europe. This trend is particularly strong for mountain territories, where such traditional agricultural practices as cattle grazing are no longer economically feasible. This study was conducted in the subalpine pasture of Cinte Tesino (TN, Italy), where local farmers have applied the following different management strategies: shorter and longer grazing durations during the season and a complete abandonment for the last 15 years. We aimed to study how these different management strategies impact the functioning and diversity of vegetation and the chemical and biological characteristics of the soil. Species richness was higher in plots subjected to longer grazing with a prevalence of *D. caespitosa* in terms of biomass share. A decline in species richness in abandoned plots was accompanied by an increase in the share of other graminoids in collected biomass. A concomitant increase in leaf N concentration and light availability in grazed plots resulted in higher photosynthetic efficiency in some species, as revealed by the δ^13^C of plant tissues. Soils under grazing were characterised by a higher concentration of total and extractable N, almost doubled microbial biomass C and increased extracellular enzymes activity, evidencing nutrient cycling mobilization. While the microbial pool was characterised by lower mineralization rates, C was lost from the soil with 15 years of abandonment. The longer grazing season demonstrated to be the most beneficial, promoting species richness, C accumulation and better soil microbial functioning. A change in soil pH from strongly acidic to moderately acidic with longer grazing is likely one of the important factors adding to the success in the functioning of primary producers and decomposers in this site.

## 1. Introduction

Alpine and sub-alpine seminatural grasslands have been formed and shaped through millennia by human agricultural activity coupled to particular mountain climatic conditions [1]. According to some studies, people initiated cattle grazing in European alpine areas starting from the early Neolithic period [2]. Traditional alpeggio (herd displacement to high-altitude pastures in the summer period) is executed by local farmers and guarantees the existence of these productive ecosystems below the tree line. Cultural traditions, morphology and climatic heterogeneity place seminatural grasslands in the Alps among the ecosystems with the highest biodiversity [3,4,5]. The European Habitat Directive (92/43/EEC) lists these ecosystems among its priority habitats. Abandonment of pastures and instauration of secondary forests reduces the biodiversity of these areas [6]. In the last century, owing to rapid industrialization and economic growth, the agricultural sector in Europe has exhibited fundamental changes [7]. The most pronounced change concerns the Alps and Pyrenees mountain areas due to difficulties in mechanization and less profitability compared to lowlands [8,9,10]. Traditional practices of mountain grasslands management (e.g., alpeggio and hay production) have been substituted with intensified farming in the lowlands and valleys [11]. As a result, the area covered by seminatural grasslands has gradually reduced, giving space to forest recolonization through a secondary succession [8,12]. Due to widespread land abandonment and changes in land use, landscape diversity associated with grasslands biodiversity is progressively reducing in the European Alps [5].

Estimates suggest grasslands store 343 Pg of C in the topsoil (i.e., top 30 cm of mineral soil), which exceeds C storage in the forest soils [13,14]. On the one hand, a high percentage of mineral-associated organic matter in grassland soils ensures major protection and stability compared to forest soils [15]. On the other hand, such soil characteristics also define the saturation limit for soil organic carbon (SOC) sequestration in grasslands [15,16]. Still, a considerable amount of SOC in grasslands appears to be sensitive to perturbations or natural changes (e.g., secondary succession), leading to SOC losses. Numerous studies have reported a net loss of SOC due to the conversion of grasslands to croplands [17,18] or due to secondary succession after grazing abandonment [19,20]. However, increases in soil C in conversion from pasture to secondary forest have also been reported [21,22]. This inconsistency could be related to management history, to its intensity [23,24] or to the climatic conditions of the researched territories [25].

Grazing has been signed by a 4 per mile Initiative launched at the COP21 among the best management practices supporting SOC accumulation and storage [26]. A comparison of the full greenhouse gas (GHG) balance between European grasslands suggested extensive pastures to act as C sinks [27]. Mechanisms of efficient C sequestration in grassland ecosystems are likely linked to the strong coupling between the elemental cycles of C and N through plants and the soil microbial functioning and diversity [28]. Grazing introduces a disequilibrium in these systems. Sustainable grazing can increase the photosynthetic potential of alpine plants through N inputs and light availability [29,30] and promote C sequestration [24,31]. The overgrazing instead will have negative consequences for vegetation productivity, GHG balance, biodiversity and SOC levels [32,33]. Practices that farmers can adopt at the local level to improve C–N coupling include, among others, improved seasonal grazing with multiple isolated patches and the maintenance of multiple grazing intensities to support biodiversity, better animal breeds and improved feed conversion efficiency [28]. Alpeggio, a traditional seasonal grazing method in mountain areas, already includes many of these features. However, studies assessing the effects of alpeggio and its abandonment on vegetation and soil properties are not numerous.

The aim of this study was to assess the state and diversity of the primary producers and decomposers under traditional alpeggio executed in a subalpine grassland in the Trentino region in Italy at different seasonal durations: grazing pressure of 1.0 ca ha^−1^ concentrated in two months (LG), grazing pressure of 0.6 ca ha^−1^ concentrated in one month (SG) and a complete abandonment for 15 years (NG). Links between aboveground and belowground processes and their interactions with management methods was the primary focus of this study. Particular attention was given to (1) tracing changes in vegetation diversity with management variation and determining the driving factors, including variation in soil characteristics; (2) assessing vegetation functioning and its relation to variation in nutrient quality and supply under different management methods and (3) tracing changes in microbial functioning and their relation to the quality of the organic matter, emphasizing possible consequences for C sequestration potential.

## 2. Results

### 2.1. Vegetation Diversity

**Figure 1:**
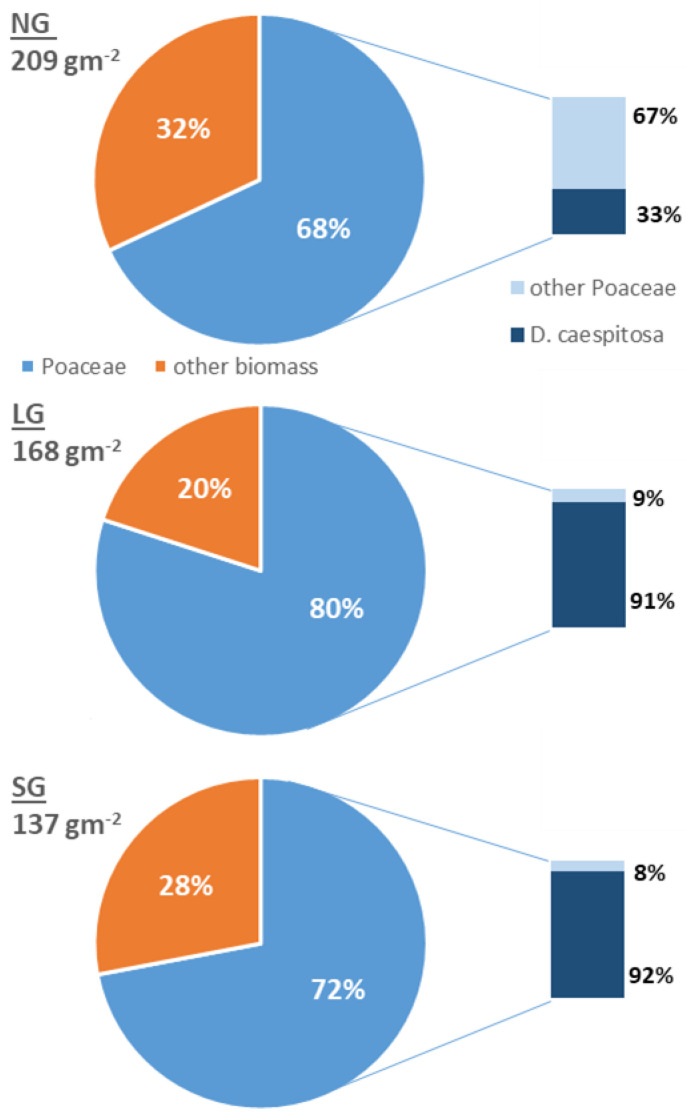
Percentage contribution of graminoids to total biomass and fractions of *D. caespitosa* in graminoid biomass. Total biomass is given in black.

Graminoids were the most abundant group in terms of biomass in all three studied grazing regimes—68% of the total mass in NG, 80% in LG and 72% in SG (*p* < 0.05). Whereas in LG and SG plots, *D. caespitosa* had absolute dominance within the detected graminoid species; in NG plots it declined from 90% to 30% of total graminoid biomass (*p* < 0.05, Figure 1). In NG plots, an increase in the fraction of nitrophilous species, adapted for degraded and rural areas, and invasive species such as *Rumex acetosa* L., (18%), *Urtica dioica* L. (5%) and *Hypericum perforatum* L. (5%) was recorded (Table 1).

In terms of the species richness, it was significantly higher in LG plots in comparison to SG and NG, where an increase in the number of non-graminoid species was registered (Table 1). In terms of the Shannon index and evenness, the three grazing regimes did not demonstrate significant differences.

### 2.2. Vegetation Functioning

**Figure 2:**
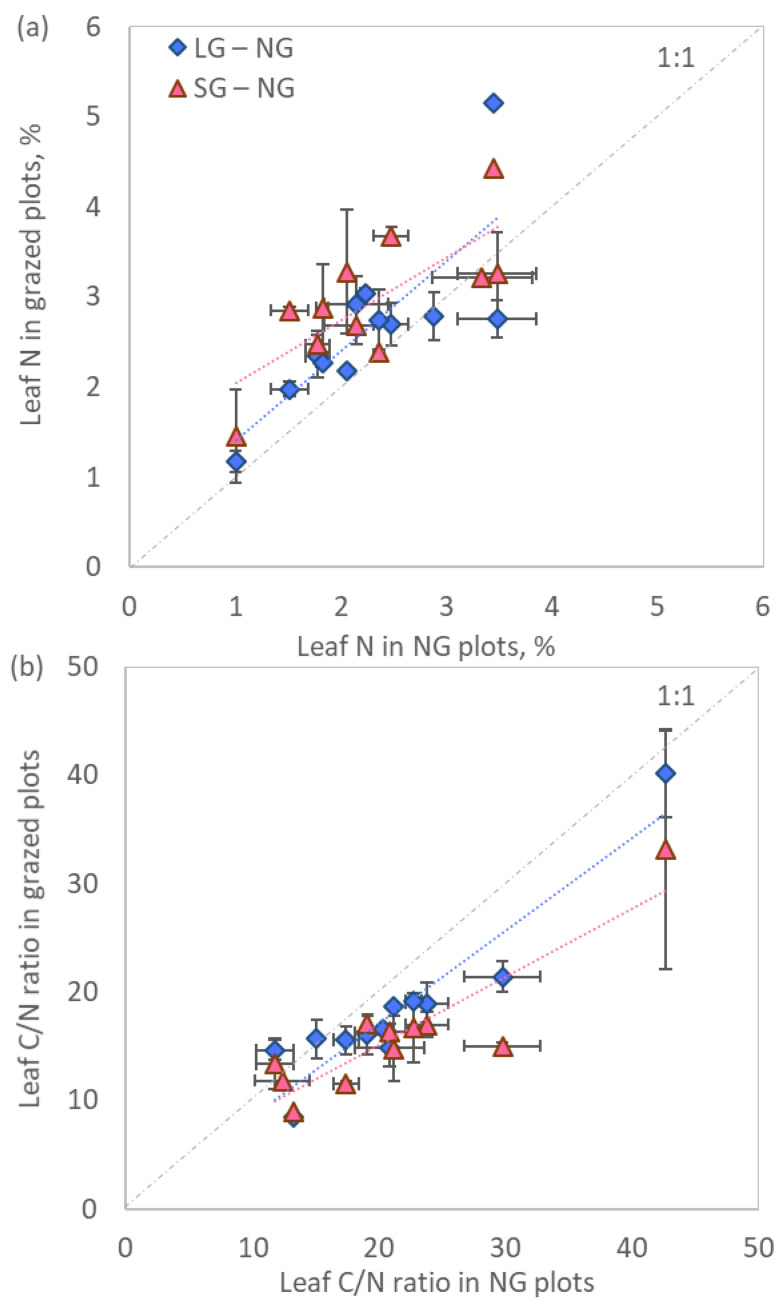
(**a**) Leaf N concentration and (**b**) leaf C/N ratio of individual species in grazed versus non grazed plots (mean ± standard error).

A common feature of many plant species was a higher concentration of N in aboveground tissues in plots subjected to grazing compared to non-grazed plots, which determined a lower C/N ratio of these species (Figure 2). Plants under the SG regime tended to have more N and a lower C/N ratio with respect to those growing under LG grazing, although differences were rarely significant (Table 2).

Among the species chosen as bioindicators, *D. caespitose*, *Festuca sp*. and *A. vulgaris* significantly increased the N concentration of the green tissues in SG plots in comparison to NG (Table 2). Two other species, *A. millefolium* and nitrophilous *R. acetosa*, did not show any changes in N concertation or C/N ratio among different grazing regimes. At the root level differences in N, concentrations were smoothed (Table S1).

The isotope composition of green tissues demonstrated that two species, *A. millefolium* and *A. vulgaris*, were characterised by a significantly higher δ^13^C of plant material under grazing (δ^13^C_veg_) (Table 2). These differences were maintained at the root level (Table S1). A similar trend, although not significant, was found for two graminoid species. In terms of vegetation δ^15^N (δ^15^N_veg_), there were no significant differences between the grazing regimes among species; moreover, δ^15^N_veg_ was characterised by large variability among replicate plots for green tissues and roots.

### 2.3. Soil Chemistry and Biochemistry

**Figure 3:**
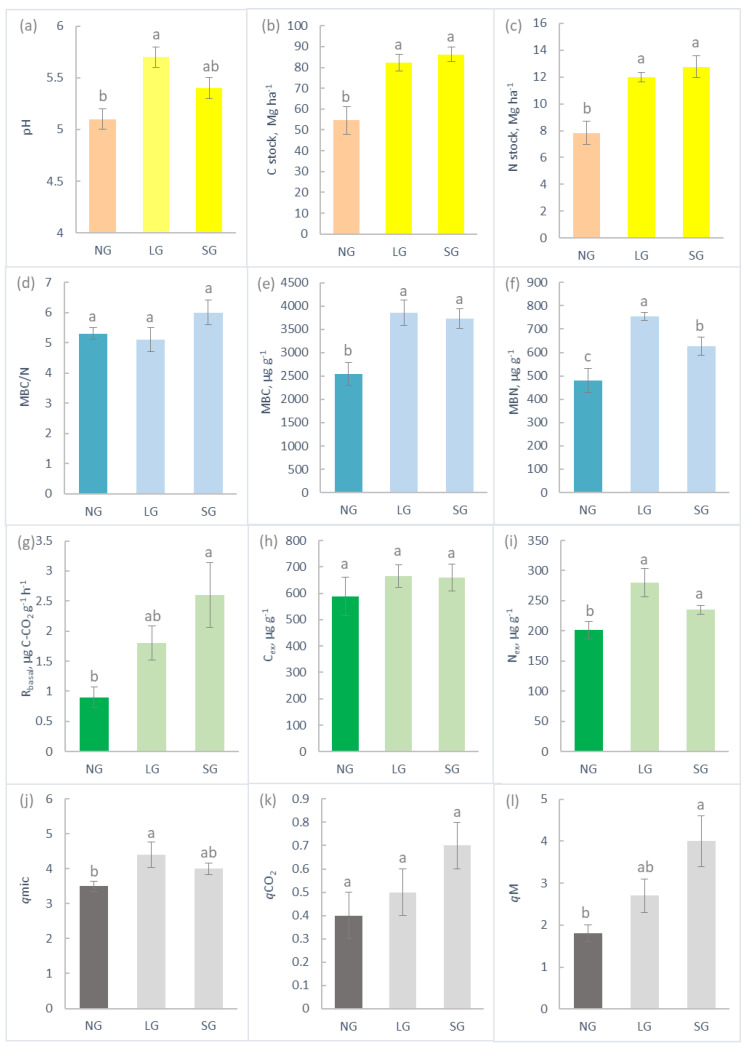
Soil chemical and biochemical characteristics measured in NG, LG and SG plots (mean ± standard error): (**a**) pH in water solution; (**b**) soil C stock; (**c**) soil N stock; (**d**) C/N microbial ratio (MBC/N); (**e**) microbial biomass C (MBC); (**f**) microbial biomass N (MBN); (**g**) microbial basal respiration (R_basal_); (**h**) soil extractable C (C_ex_); (**i**) soil extractable N (N_ex_); (**j**) soil microbial quotient (*q*mic); (**k**) soil metabolic quotient (*qCO*_2_); (**l**) soil mineralization quotient *(q*M*).* Letters next to the bars show the significance of ANOVA at *p* < 0.05.

**Figure 4:**
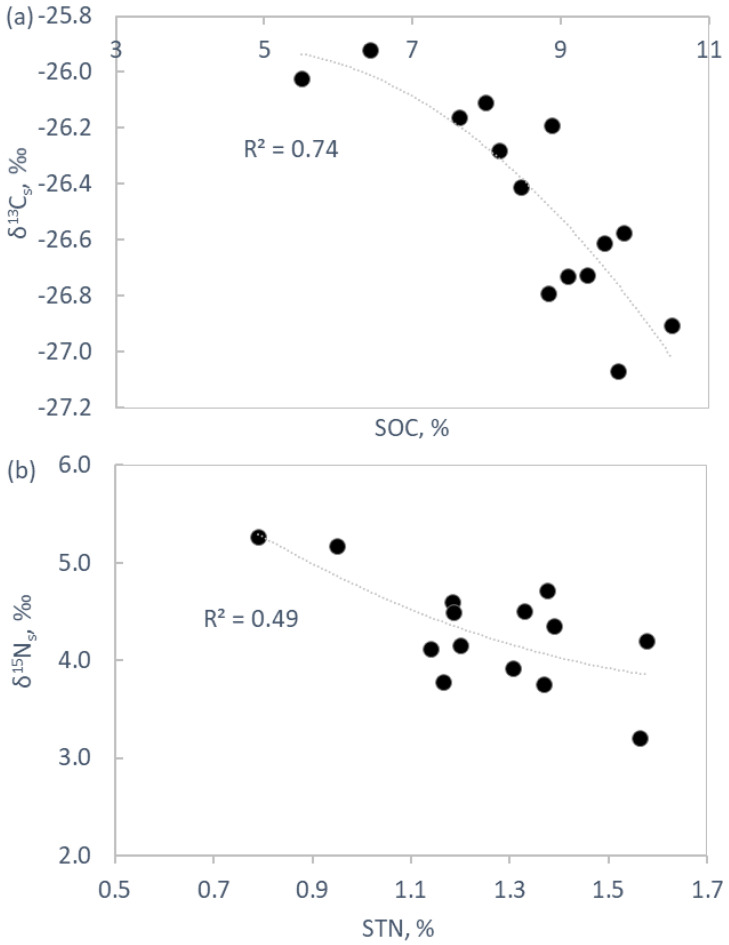
(**a**) Soil δ^13^C (δ^13^C_s_) plotted versus soil organic C concentration (SOC); (**b**) soil δ^15^N (δ^15^N_s_) plotted versus total soil N concentration (STN).

**Figure 5:**
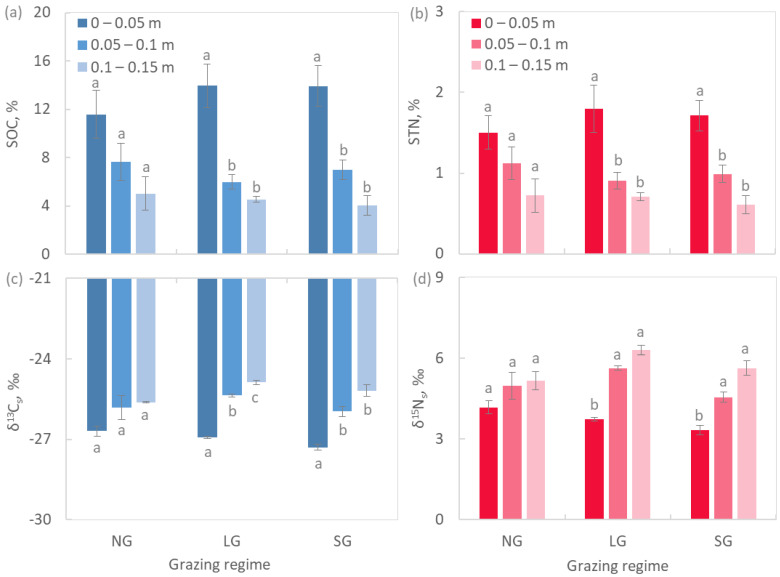
Depth distribution of (**a**) soil organic C (SOC); (**b**) total soil N (STN); (**c**) soil δ^13^C (δ^13^C_s_) and (**d**) soil δ^15^N (δ^15^N_s_) in grazed and non-grazed plots (mean ± standard error). Letters next to the bars show the significance of ANOVA at *p* < 0.05.

Soil pH varied from moderately acidic in LG plots to strongly acidic in NG and SG plots (Figure 3a). Soils in grazed plots were characterised by a higher C and N stocks (Figure 3b,c). SOC concentration was inversely related to its δ^13^C (δ^13^C_s_), once all replicate data were pooled together (*p* < 0.001, Figure 4). Soil total N (STN) concentration was negatively correlated to its δ^15^N (δ^15^N_s_) (*p* < 0.01, Figure 4). The first 0.15 m of soil were characterised by major homogeneity in terms of SOC, STN, δ^13^C_s_ and δ^15^N_s_ vertical distribution in NG plots, whereas in grazed plots, the first 0.05 m significantly differed in these variables with respect to the underlying layers (Figure 5).

Both MBC and MBN were 1.5 times higher in grazed plots compared to NG plots (Figure 3e,f), resulting in similar microbial C/N ratios among the two grazing regimes (Figure 3d). The microbial quotient (*q*mic) was also higher in grazed plots, although the differences between values for NG and SG were not significant (Figure 3j). While the amount of C_ex_ was similar between the grazing regimes, the amount of N_ex_ was significantly higher in soils under grazing compared to NG (Figure 3h,i).

Microbial basal respiration, R_basal_, was significantly higher in SG plots compared to NG and was not different from LG and between LG and NG (Figure 3g). R_cum_ was characterised by the same pattern (Figure S1, Supplementary Materials). R_cum_ and R_basal_ determined the patterns of two ecophysiological indices calculated on their bases: *q*M and *q*CO_2_, respectively (Figure 3k,l).

### 2.4. Extracellular Enzymes 

**Figure 6:**
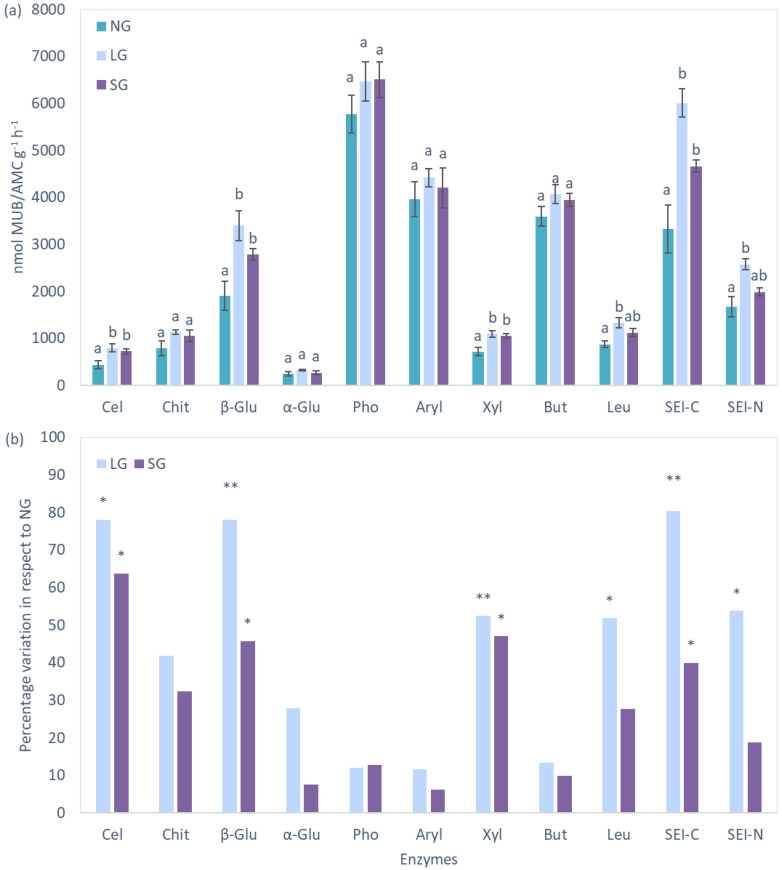
Soil extracellular enzyme activities and SEI index for enzymes involved in C and N cycles in grazed and non-grazed plots (**a**) absolute values (mean ± standard error), Letters next to the bars show the significance of ANOVA at *p* < 0.05; (**b**) percentage change in respect to non-grazed plots, significant differences with NG plots are indicated by * at *p* < 0.05 and ** at *p* < 0.01. Cel = β-cellobiohydrolase; Chit = Chitinase; β-Glu = β-glucosidase; α-Glu = α-glucosidase; Pho = acid phosphatise; Aryl = arylsulphatase; Xyl = β-xylosidase; But = butyrate esterase; Leu = leucine-aminopeptidase; SEI-C and SEI-N = synthetic enzymatic index of C and N enzymes.

**Figure 7:**
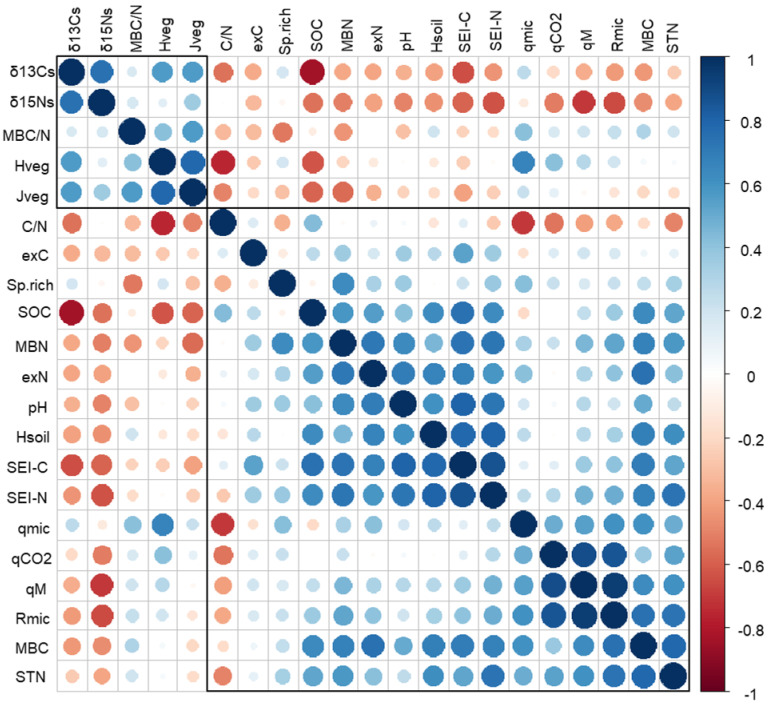
Spearman correlation matrix. Data are clustered according to the similarity in patterns of correlation coefficients.

**Figure 8:**
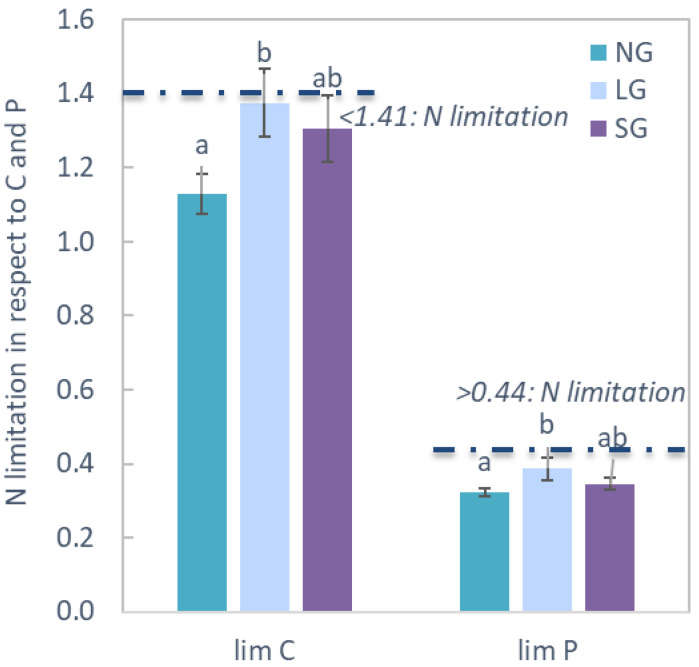
Soil N limitation with respect to C (lim C) and P (lim P) determined as the ratio between activities of enzymes of N cycle with β-glucosidase and acid phosphatase, respectively (mean ± standard error). Letters next to the bars show the significance of ANOVA at *p* < 0.05. Dashed lines indicate the limitation threshold of N in respect to C and P.

Extracellular enzyme activity was accelerated in grazed plots for six out of the nine evaluated enzymes (Figure 6). Because C and N cycling were the most affected, the related synthetic enzymatic indices (SEI-C and SEI-N) were significantly higher in grazed plots. The best variables to describe the variation in SEI-C were SOC (R^2^ = 0.66, *p* < 0.001), N_ex_ (R^2^ = 0.57, *p* < 0.01), MBN (R^2^ = 0.62, *p* < 0.01), MBC (R^2^ = 0.55, *p* < 0.01) and soil pH (R^2^ = 0.54, *p* < 0.01) (Figure 7). The strongest correlations for SEI-N were found for soil δ^15^N (R^2^ = 0.57, *p* < 0.01), STN (R^2^ = 0.60, *p* < 0.01) and MBC (R^2^ = 0.54, *p* < 0.01). Potential limitation for N with respect to C was more pronounced in NG plots, whilst N limitation in respect to P availability was higher in LG plots, although not exceeding the limitation threshold (Figure 8).

When the enzymatic activity was expressed per unit of MBC, it levelled between grazed and NG plots, with the exception of acid phosphatase, arylsulfatase and butyrate esterase, which declined with grazing (Figure S2a, Supplementary Materials). Once expressed per unit of SOC, specific enzymatic activity was higher in grazed plots for enzymes involved in the C cycle (β-glucosidase, β-xylosidase, β-cellobiohydrolase) and N cycle (leucine aminopeptidase) (Figure S2b, Supplementary Materials). H_soil_, calculated using enzyme activity data, did not show any significant difference between evaluated management regimes (data not shown).

## 3. Discussions

### 3.1. Biodiversity

Our survey was carried out at the end of the grazing period. Animal N inputs, urea and faeces have increased the soil N pools over time, benefiting plant species with high nutritional needs (Table 1 and Table 2). A general dominance of nitrophilous and resistant species such as *D. caespitosa* was observed. A significantly higher number of species and a mosaic species distribution were found in pasture under prolonged grazing (LG). Abandonment of grazing was reported to negatively impact species richness [34,35]. Moreover, a clear association between high species richness and the management of seminatural grasslands has been reported [34,36]. In our study site, abandonment promoted competitive grasses, which formed a dense cover on the site, and invasive and synanthropic species such as *Rumex acetosa* L. (18% of mass), *Urtica dioica* L. (5% of mass) and *Hypericum perforatum* L. (5% of mass). Among the mechanisms behind the loss of species richness with abandonment, it should be mentioned that an increase in the biomass of certain species outcompete the development of others in competition for nutrients and, even more so, for light [37]. While grazers increase nutrient availability to vegetation, they also regulate light availability and rescue diversity. Based on these criteria, the longer the grazing duration, the longer it executes a light-regulating function.

In comparison with the other treatments, LG soils were characterised by higher pH values. Generally, the relationship between species richness and soil pH is described as linear [38]. Grasses are considered among the species resistant to acidification [39]. Values measured for LG correspond to the optimum values established for mountain grasslands and may contribute to the greater species richness and increase in the fraction of non-graminoids observed in this management type [40].

The absolute dominance of *D. caespitosa* over other forage grasses in grazed plots is likely linked to a failure to adapt pasture management to the earlier growing season onset. Progressively warmer springs, shorter snow duration and earlier leaf out have been observed for these elevations in recent decades [41]. When animals are brought to the pastures in mid-June, most species are already at the maturity stage and, in the case of *D. caespitosa*, already at very low forage value, so the plant is avoided by the livestock [42]. Forecasts of weather conditions and adjustment of the grazing starting period may improve vegetation diversity on the one hand and increase herbage digestibility and productivity output on the other [43]. Furthermore, the site was previously managed through seasonal mowing. The reintroduction of this practice may help to balance the species composition of the pasture [44]. While grazing was found to give more benefits in terms of biodiversity conservation, mowing can promote different grazing plant traits, such as a further decrease in inter- and intraspecific competition, and support the growth of numerous species at a small scale [45].

**Figure 9:**
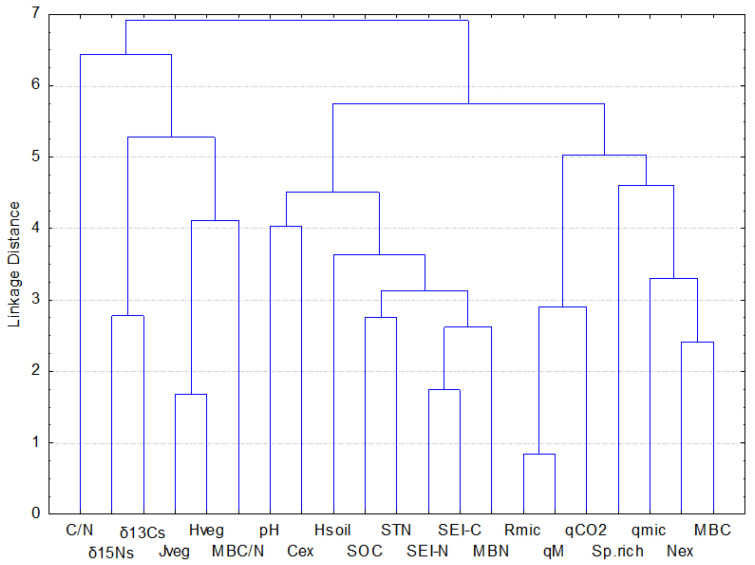
Joining tree clustering analyses of soil and vegetation-related variables.

Variables linked to vegetation diversity, H_veg_ and J_veg_, formed a separate cluster with such soil variables as MBC/N and C/N ratios and the C and N isotope composition of soil (Figure 9). Clustering with soil C/N ratios, δ^13^C_s_ and δ^15^N_s_ can be explained by a common vegetation origin of these variables, and the link is likely enhanced through the grazing loop [46]. The MBC/N ratio is instead used as an indicator of fungi and bacteria abundance in soils because of the different C/N ratios of the cells [47,48]. Vegetation diversity has many possibilities to impact bacteria and fungi succession through exudates and litter quantity and quality, making the MBC/N ratio more vegetation-related than soil-related [49,50].

Functional diversity of the microbial community, H_soil_, was not affected by variation in the management regime the way it was with H_veg_. Furthermore, H_soil_ did not correlate with H_veg_ and formed a separate cluster with soil chemical and biochemical variables that do not confirm an observation of similar patterns between these two variables, as reported in some studies (Figure 7 and Figure 9) [51,52]. Still, we did find a strong and inverse relationship between microbial diversity and species richness within the LG grazing regime, characterised by major variability in the number of species among plots. Here, plots with the highest species richness were characterised by the lowest H_soil_ (R^2^ = 0.92, *p* < 0.01, Figure S3a, Supplementary Materials) and lowest SEI-C (R^2^ = 0.92, *p* < 0.05, Figure S3b, Supplementary Materials). This corroborates the finding that microbes and plants could be linked at different levels of diversity organization [53,54]. H_soil_, calculated by means of enzymatic activities, represents the sum of ecological processes or the capacity to use different substrates developed by the organisms of a community. It may be enhanced either by the necessity of microbes to obtain essential nutrients (e.g., under oligotrophic conditions) or to degrade diverse, chemically complex substrates [55,56,57]. In this study, it may be hypothesised that an increase in grass species richness, found under LG, promoted the release of a great variety of root exudates, which in grasslands are known to be generally constituted by low molecular weight compounds [58,59]. An increase in root exudation is also a known response of vegetation to grazing [60,61]. The access to easily degradable substrates may have repressed the range of enzymes generally involved in the degradation processes of recalcitrant substrates. A decline in the number of genes involved in the cycling of C under grazing has been reported for alpine grasslands [51].

### 3.2. Vegetation Functioning

The general trend for species grown in grazed patches was an increase in the amount of leaf N and a consequent decline in the C/N ratio. This reflects a higher availability of N in grazed patches, as confirmed by a higher amount of N_ex_ and total N in these soils. On the one hand, the increase in N concentration of tissues makes the vegetation more palatable for grazers. On the other hand, it ensures a faster regrowth rate, makes this material more accessible for decomposers and accelerates the nutrient cycling and release of substances for growth of microbial biomass and vegetation regrowth, thereby completing a grazing loop [46,62]. Plants under grazing are generally fast-growing and benefit from N to recover tissues damaged by defoliation, thus increasing grasslands productivity [63,64].

Among five indicator species, a significant increase in the N concentration of tissues was measured in three of them (*D. caespitosa, Festuca sp.* and *A. vulgaris*). In *D. caespitosa* a strong and inverse relationship between the tissues’ N concentration and ^15^N composition of the soil in plots was observed (R^2^ = 0.51, *p* < 0.01, Figure S4, Supplementary Materials). A similar pattern, although not significant, was observed for *A. millefolium* (R^2^ = 0.28, ns) and *A. vulgaris* (R^2^ = 0.27, ns). The release of ^15^N depleted bovine urine into the soil affects the soil’s δ^15^N [65,66]. Urine is the main source of N in pastures, although a major part of it could be lost through volatilization and leaching [67]. Another source of N is bovine faeces. Being less subjected to losses in comparison to urine, its contribution to soil N mobilization could be substantial [68,69]. Faeces sampled from the study area were characterised by quite low δ^15^N, thereby contributing to the lowering of the soil’s δ^15^N. The return of C and N to soil with animal excreta is not homogenous though, and hotspots with high nutrient availability are created [70]. An inverse correlation of plant N concentration with the ^15^N composition of the soil supports a nonhomogeneous distribution of nutrients and access to depleted N forms in the hotspots. Indeed, plants possess all the necessary mechanisms for rapid and direct uptake of urea and could even bypass its microbiological conversion to more simple inorganic compounds [71].

*A. vulgaris* and *A. millefolium* increased their biomass in grazed plots (Table 2). This increase was accompanied by an increase in the δ^13^C_veg_ of the green tissues. It is well known that δ^13^C_veg_ is considered a valuable proxy for time-integrated intrinsic water use-efficiency (iWUE), i.e., the ratio between CO_2_ assimilation rate (A) and stomatal conductance (gs) [72]. Hence, under conditions of nonrestricted soil water content, an increase in the δ^13^C of plants growing in grazed plots could indicate a higher A/gs in comparison to plants growing in NG plots, due to a higher carboxylation efficiency [73,74]. Indeed, a concomitant increase in N concentration suggests that the N in excess is likely invested in the photosynthetic apparatus (e.g., rubisco). However, improvement in the light conditions leading to a higher photosynthetic capacity should be also considered [37].

### 3.3. Soil

The shifts in vegetation biomass and diversity at Malga Arpaco with the change in management strategy are derived either from direct (e.g., grazing) or indirect (e.g., soil properties) factors. Grazing, and its relative intensity, can in fact induce significant changes in soil properties caused by multiple factors acting in different directions, including a reduction in aboveground litter and a change to its quality [63], an increase in quantity and quality of root exudates [75], modification of the soil’s physical environment (e.g., moisture, temperature, aeration and structure) through trampling [76,77] and fertilization of soil through animals’ excreta [69]. These changes may impact SOC stocks [23,25], alter nutrient cycling (especially N) and ultimately modify other soil abiotic factors including pH and organic matter availability, which, in turn, drive microorganisms’ biomass, activity rate (i.e., function) and diversity [78,79,80].

SOC concentrations at Malga Arpaco were consistent with estimates for grasslands in similar climatic conditions [81,82,83]. As for N, the STN concentrations were considerable when compared with the abovementioned studies or studies performed on temperate grasslands [84,85]. This was evident even in plots where grazing had been interrupted over the previous 15 years. The difference can be explained by slowed biogeochemical cycling, larger immobilization of N into stabilised humic forms into soils not disturbed by grazing [86] and, lastly, to the short time passed since grazing interruption. Still, N limitation in comparison to C availability was more pronounced in abandoned plots, as indicated by the decline in the ratio of β-glucosidase to the sum of N-related enzymes (Figure 8). Values of this ratio lower than 1.41 indicate a microbial requirement of N sources with respect to C [87].

Animal excreta is the primary factor impacting soil N concentration under grazing because urea is highly soluble and is rapidly taken up by plants [69,71,88]. Animal faeces also contribute to replenishing soil nutrient pools, but their decomposition proceeds more slowly than for urea and its effects on vegetation are less immediate [89]. Therefore, although C and N are withdrawn from the system through animals’ uptake and part of it is lost, these elements return to the soil in more accessible forms through animal excreta. This is particularly evident in surface layers, which accumulated less stable C and N forms that are likely associated with the animals’ excreta (Figure 5). Such abrupt changes in the quality of organic matter with soil depth were not found in NG soil. 

The stable isotopes analysis revealed the inverse significant relationships of SOC with δ^13^C_s_ and of STN with δ^15^N_s_ (Figure 4). The depletion of soil in heavy isotopes under grazing could be due to the less decomposed state of organic matter and because of the input of isotopically lighter material to the upper soil layers. Microbial degradation processes promote a gradual enrichment in ^15^N and ^13^C of the organic matter, trends typically observed with an increase in soil depth [90,91]. However, increases in the mineralization quotient (*q*M) and specific enzymatic activities (enz SOC^−1^) do not support the less decomposed organic matter hypothesis for soils under grazing and, in fact, suggests exactly the opposite. These two variables are also used as indicators of the substrate quality and, hence, point to different qualities of soil organic matter between grazed and NG soils. Ureic N may be the first cause of soil isotopic differences because animals fix ^15^N into tissues more easily and release lighter isotopes (from −1 to −4 % with respect to the diet) with their urine [65]. The δ^13^C observed in faeces (δ^13^C: −28.65 ± 0.12) is in accordance with a study that reported a depletion in ^13^C in bovine faeces with an average fractionation of −0.8% with respect to diet [92]. The δ^13^C observed in faeces (δ^13^C: −28.65 ± 0.12) is in accordance with a study that reported a depletion in ^13^C in bovine faeces with an average fractionation of −0.8% with respect to diet. The observed δ^15^N values of faeces (Table 3) were lower than previously reported [93], likely because of NH_3_ volatilization limitations due to acidic soil pH [94].

The increase in microbial biomass, enzymatic activities and microbial basal and cumulative respiration indicates stimulation of the nutrients’ mobilization process in grazed plots. Acceleration of nutrient cycling with grazing was previously reported [78,80,95]. The N sources made available through the animals’ fertilization and C substrates through plants increased the release of exudates in response to defoliation [60], thereby boosting microbial activity and nutrient cycling. Furthermore, alkalinisation of acid soils through depositions of animal excreta and urea degradation [96] stimulates the SOC mineralization rate and the release of soluble C and N forms [97]. These processes promote microbial biomass and are more specialised in the use of easily degradable resources, with lower efficiency in the use of C resources, as suggested by the observed increase trend in the microbial metabolic quotient (*q*CO_2_) from abandoned to managed soils. 

However, despite the higher mineralization rates and lower C use efficiency with respect to abandoned plots, the SOC and N balance was positive in the grazed plots: C and N concentrations and stocks were higher in grazed plots in confront to the abandoned one. This was also confirmed by the increase in another ecophysiological index, the microbial quotient (*q*mic), which is considered an early predictor of changes in soil organic matter. We can hypothesise that *q*mic was larger in LG due to more prolonged grazing activity during the year, favouring a slow stabilization or a steady state of the microbial community. Hence, we can confirm the further potential for C accumulation for Malga Arpaca soils under grazing. It should be considered, however, that positive effects of grazing on SOC accumulation are strongly related to climate zones [25]. 

As for the soluble forms of C and N, significant changes were observed only for N, which increased under grazing. Although it is known that grazing promotes the growth and renewal of plant root systems [95] and increases the flux of rhizodepositions to soil [75], we may hypothesise that the extra C substrates were immediately respired or immobilised by microbes, given the contemporary availability of soluble N, promoting further stabilization of organic matter. 

Differences in soil biochemical properties between two types of grazing: short and long, may be ascribed by the unsteady situation stressing microbial metabolism in shorter grazing. A lower load of animal excreta left soil pH at more acidic values, lowered the amount of extractable N and MBN and contributed to an increase in the metabolic quotient in these soils and a decline in the activity of some enzymes in comparison to the LG regime.

## 4. Materials and Methods

### 4.1. Site Description

**Figure 10:**
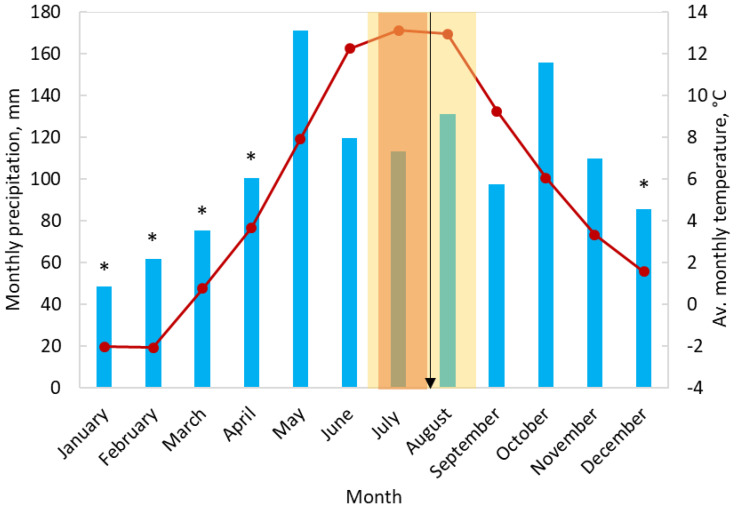
Monthly precipitations and average monthly temperatures measured in meteorological station of Malga Arpaco in the period 2003–2017. Yellow area refers to the grazing period, typically applied for LG patch; orange—for SG patch, arrow indicate a sampling period, “*” indicate months with snow precipitation.

The experimental site, Malga Arpaco (MA, 46°06′50″ N 11°42′13″ E), is located 1730 m above sea level, close to Passo Brocon in the Trentino region in Italy. The pastures are surrounded by coniferous forests and belong to the nearby municipality of Cinte Tesino. Pasture management is carried out by farmers who rent these lands from the local authorities. The climate of the area is mountainous, with an average annual temperature of 5.4 °C and annual precipitation varying from 1100 mm to 2000 mm for the period 2007–2017 (Figure 10). Maximum snow depth amounts on average to 760 mm and is reached typically in February. Snow is generally completely melted between April and May.

Natural subalpine vegetation, pure or mixed spruce and larch forests, typical for these altitudes, is limited in the area of study to the steep slopes. Human activities have deeply modified the landscape, which is still dominated by meadows and pastures. The depth of the soil in the site varies on average from 0.15 to 0.3 m depending on location. The soil is classified as an Alfisol with clay horizon derived from leaching, rich in silicates and ferrous compounds [98]. Soil texture in the first 0.30 m is clay loam with 42% silt, 37% clay and 21% sand.

The onset of grazing starts typically in mid-June. Patches of grassland are delimited with electric fences and the animals graze the patches for a restricted time period, typically from one to two months, and are then moved to other patches. Seasonal grazing terminates between the end of August and the beginning of September, when the animals are brought back to the valleys. The study was concentrated in three patches characterised by different grazing pressures. One of the patches was characterised by a grazing pressure of 1.0 ca ha^−1^ concentrated in two months: long grazing (LG). Another patch was characterised by a grazing pressure of 0.6 ca ha^−1^ concentrated in one month: short grazing (SG). The number of cattle present in the patches per hectare during these periods was essentially the same; the difference in the grazing pressure was obtained through the different grazing durations. These two patches have been historically grazed and the reported grazing regimes can be traced back, at least, for the previous six years. The LG and SG patches have a dimension of approximately 7 ha and are located in close proximity to each other, separated by a strip excluded from grazing in the last 15 years (NG patch). The dimension of NG patch is 0.05 ha. The intake forage rate was estimated by the herbal disappearance method in the year prior to the current extensive sampling using a battery-powered hand clipper and collecting the biomass in 8 replicated plots of 1.2 × 0.07 m size in LG and SG patches [99]. Sampling was carried out prior to the grazing onset and one month after its initiation; hence, the biomass regrowth during this period was not considered. The intake rate for both patches amounted on average to 7 g m^−2^ d^−1^.

### 4.2. Sampling

Plant and soil sampling was performed in the second half of July 2018. Similar to many ecological studies, our experimental design is a variant of the “real situation”, where pseudoreplication is often an unavoidable issue [100]. We could not regulate the stock presence, nor could we introduce additional fences. The consideration of another farm in the surrounding area would inevitably change the stock density, elevation and soil type and would not serve as a solution. We are aware that our experimental setup is based on one area of fenced-in land and, hence, we cannot completely exclude the possibility that the measured differences in variables of interest are driven by other factors rather than management intensity and abandonment. Still, we tried to minimize this possibility. Plots in LG and SG patches were established at the side of NG patch to form a transect, considering a 10 m buffering distance from NG and a 10 m distance between each replicated plot. The buffering distance from the fence was maintained for NG plots as well. Such precautions are important in order to minimize possible confounding effects of background conditions with management treatments and to ensure similar elevation, exposition as well as soil and hydrological conditions [101].

After a botanic survey, the aboveground biomass of each species present in 1 m^2^ plots was separately collected. Roots were subsampled, carefully cleaned from soil in the isotonic solution, freeze-dried and stored until analyses. Plant material sampled for determination of Shannon–Weaver and Evenness indices was oven dried at 60 °C until it reached a constant weight. A subsample for biochemical analyses was freeze dried. 

Among the analysed species, as bioindicators of vegetation changes induced by different management methods, the following species, which were present in all three grazing regimes, were chosen: *Deschampsia caespitosa* (L.) Beauv., *Festuca* sp., *Achillea millefolium* (Asteraceae family) L., *Alchemilla vulgaris* L. (Rosaceae family) and *Rumex acetosa* L. (Polygonaceae family). At the time of sampling, it was not possible to characterise plants of genus *Festuca* up to the species level. This group may include plants of *Festuca pratensis*, *Festuca varia* and *Festuca rubra* as demonstrated by the previous botanic surveys of the site.

Mineral soil was sampled to a depth of 0.15 m at four corners and in the centre of the plot and combined in one composite sample. Separately, samples were taken for the bulk density determination: a known volume of soil was sampled for the depths 0–0.05, 0.05–0.1 and 0.1–0.15 m. Soil bulk density of different layers was averaged for C and N stock estimation. The depth of sampling was considered representative for this shallow soil. 

### 4.3. Isotope Characterization of Vegetation 

From aboveground biomass, only green tissues were selected for stable isotope analyses because species were in different phenological phases at the time of sampling; reproductive and lignified parts were not considered. The dried plant material was ground to a fine powder and an aliquot (~0.2 mg) of each sample was used to determine the concentration and isotope composition of C and N (δ^13^C_veg_, δ^15^N_veg_) using an IRMS (Isoprime, Cheadle, UK) coupled to an elemental analyser (NA1500, Carlo Erba, Milan, Italy). The precision of isotopic measurements was determined against two IAEA and one internal standard for each considered element and was better than 0.1‰. In particular, NBS-22 oil, IAEA-CH6 sucrosec and IAEA-600 caffeine were used for scale normalization of measured δ^13^C values to the VPDB scale. For δ^15^N_veg_, we used IAEA-600 caffeine, USGS40 glutamic acid and IAEA-NO-3 KNO3.

### 4.4. Soil Chemical and Biochemical Analyses

The following soil characteristics were determined: organic carbon (SOC) and total nitrogen (STN) concentrations and their isotope composition (δ^13^C_s_ and δ^15^N_s_), C and N stocks, microbial biomass carbon (MBC), microbial biomass nitrogen (MBN), extractable carbon (C_ex_), extractable N (N_ex_), basal respiration (R_basal_), cumulative respiration (R_cum_) and extracellular enzymes involved in the cycling of C, N, P and S. Air-dried soil was sieved at 2 mm and carefully cleaned from roots. Part of it was further dried at 105 °C until there was a constant weight for SOC, STN concentration and related stock determination. SOC, STN and their isotope compositions were determined after removal of the carbonates with 10% HCl [102]. Measurements were carried out on an IRMS (Isoprime, Cheadle, UK) coupled to an elemental analyser (NA1500, Carlo Erba, Milan, Italy) following protocol similar to vegetation samples. 

Another soil aliquot was used for microbiological soil characterization. This soil was air-dried before processing. MBC and MBN were determined by means of a fumigation-extraction technique [103]. Twenty grams of soil from each sample were conditioned to 60% of their water holding capacity (WHC). Half of this aliquot was fumigated with chloroform for 24 h and extracted with 40 mL of 0.5 M K_2_SO_4_; the other half was extracted without fumigation. Extracts were analysed for C and N concentration with TOC-V CSN and a TNM-1 analyser (Shimadzu, Kyoto, Japan). MBC and MBN were calculated by applying the conversion factors of 2.64 and 2.22, respectively [103,104]. The C and N concentration of non-fumigated extracts was used as a measure of easily extractable pools (C_ex_ and N_ex_). Microbial R_basal_ was measured by incubating 10 g of soil at 25 °C and 60% of WHC for 52 days [105]. The CO_2_ evolved was trapped—after one, three, seven, 10, 14, 21, 28, 35, 42 and 52 days of incubation—in 2 mL 1 M NaOH and determined by titration of the excess NaOH with 0.1 M HCl. The total CO_2_ evolved at the end of the experiment was considered as cumulative respiration (R_cum_), while average hourly CO_2_ output at the end of incubation was considered as the basal respiration (R_basal_).

Fluorogenic methylumbelliferyl (MUF) and aminomethylcoumarin (AMC) substrates were used to measure soil extracellular enzyme activity in the LG, SG and NG soils [106,107] in microplate assays. β-glucosidase (EC 3.2.1.21), α-glucosidase (EC 3.2.1.20), β-xylosidase (EC 3.2.2.27) and β-cellobiohydrolase (EC 3.2.1.91) were considered for the C cycle. For the N cycle, chitinase (EC 3.2.1.30) and leucine-aminopeptidase (EC 3.4.11.1) were chosen. Acid-phosphatase (EC 3.1.3.2) and arylsulphatase (EC 3.1.6.1) were considered for the P and S cycles, respectively. Finally, butyrate esterase (EC 3.1.1.1) was selected as a proxy of intracellular activity [108]. Fluorescence was measured with an automatic fluorimetric plate reader (Fluoroskan Ascent, Thermo Fisher Scientific, Waltham, MA, USA). Readings were taken five times—0, 30, 60, 120 and 180 min—from the start of the incubation. Limitation of N in respect to C was quantified from the enzymatic data by the ratio of β-glucosidase to the enzymes of the N cycle. Limitation of N in respect to P was considered by the ratio of enzymes of the N cycle and acid-phosphatase [109].

Together with vegetation and soil samples, fresh animal faeces were collected in the closed vicinities of each plot. Faeces were oven dried at 60 °C until they reached a constant weight and milled to a powder in a mortar. Samples were analysed for N and C concentration and stable isotope composition similar to the vegetation and soil samples.

### 4.5. Indices

Obtained data served for the quantification of a series of ecophysiological indices.

Soil microbial quotient (*q*mic) was calculated as a ratio of MBC to SOC [110]. This index is a measure of the contribution of MBC to the organic C pool of the soil; it also indicates the substrate availability to the soil microflora, representing, therefore, an early predictor of organic matter variations [104].

Soil metabolic quotient *(qCO*_2_*)* was calculated as the ratio of R_basal_ to MBC [111]. It is a measure of the efficiency of the microbial community in the utilization of the available resources (high efficiency corresponds to low values of *q*CO_2_ and vice versa); it also serves as a proxy of the stress level of the microbial community.

Soil mineralization quotient *(q*M*)* was calculated as the ratio of the R_cum_ to SOC [112]. It expresses the efficiency of the microbial community in metabolizing total organic C, or the amount of mineralized C with respect to the initial amount of SOC; it serves as an indication of the quality of the organic substances present in the soil.

The specific enzymatic activity was calculated as the ratio of each soil enzyme activity either to SOC or to MBC; this helps to highlight environmental factors, other than total organic C or microbial biomass, that may affect enzymatic activity [113]. The specific activity is also useful to discuss enzymatic activities either in relation to the quality of organic substrates (enz SOC^−1^) [113] or to the catalytic efficiency of microbial biomass (enz MBC^−1^) [114]. The synthetic enzymatic index (SEI), the sum of all enzymatic activities, was calculated as a synthetic measure of microbial functional capacities separately for C (SEI-C) and N (SEI-N) [57]. The Shannon–Weaver index (*H’*) was used to determine the soil’s microbial functional diversity [115]:

$$H^{\prime }=-\overset{S}{\underset{i=1}{\sum }}Xi/X\;\ln\;Xi/X$$

where, *Xi/X* is the ratio of the activity of a given enzyme to the sum of the activities of all the enzymes. Similar to *H’* for soil (H_soil_), indices of biological diversity were calculated for vegetation (H_veg_). Species richness was calculated by referring the number of species to the unit of surface [116], while H_veg_ was calculated using the same formula for the soil’s microbial functional diversity, with *X_i_* being the number of individuals or biomass of the species and *X* being the sum of the these quantitative variables for all the species.

To evaluate homogeneity, the evenness index (J_veg_) was calculated from the Shannon–Weaver index as [117]:

*J_veg_ = H’/ln (S)*

where, S is the species richness.

### 4.6. Statistics

Each plot was considered as an experimental replication. For soil- and plant-related variables, the mean value, standard error and variation coefficient were calculated with tools for descriptive statistics over five replicated plots for each considered management type. The Shapiro–Wilk test was used to assess data normality. One-way analyses of variance (one-way ANOVA) and Fisher post hoc multiple comparison tests were performed to assess the influence of the studied factors in all the measured variables. Clustering analyses of soil and vegetation-related variables was performed with joining tree clustering. Because these variables are characterised by different types of scales, the data were standardized prior to processing so that each variable has a mean of 0 and a standard deviation of 1. Statistical data elaboration was performed using STATISTICA software (Statsoft). The Spearman coefficient was computed between the biological and chemical characteristics of soil and vegetation, and the heat map was contracted through R studio software (RStudio, PBC, Boston, MA, USA). Data were processed with the packages “corrplot” and “ggplot2”; variables with similar patterns of correlation coefficients were placed close together and divided into a given number of groups based on hierarchical clustering. Regression analyses were performed to test the relationship between single variables of interest.

## 5. Conclusions

The primary sinks for C in grassland ecosystems are located belowground and could be subjected to saturation with time and to C losses with perturbation. Abandonment of traditional grazing activity in isolated patches of Malga Arpaco resulted in the decline in plant species diversity and loss of SOC from the topsoil. While the microbial community in the abandoned plots was characterised by a higher C use efficiency, the amount and the quality of the animal inputs allowed the C and N stocks of the grazed soils to increase. Grazing abandonment worsened the edaphic conditions by acidifying the soil, hence, negatively impacting mineralization rates and biological diversity. While traditional alpeggio is beneficial for plant functioning, biodiversity and C sequestration, an improvement in management activity can still be suggested. Such improvements may include anticipation of the grazing season facing the consequences of accelerated climate change, as there has been a progressively earlier growing season onset registered in these areas in recent decades. Anticipation can help increase the palatability of grasses such as *D. caespitosa* and level their contribution to the total biomass of grazed plots. The reintroduction of mowing and hay production practices for improving herbaceous biological diversity and elongation of the grazing period for improvement in edaphic soil conditions could also be recommended. As our study is represented by just one fence and one pasture, we acknowledge that the observed differences in vegetation and soil-related variables are specific for just our site and any generalization should be made with care.

## Figures and Tables

**Table 1:** Relative abundances of the botanical families in NG, LG and SG plots calculated on the basis of species biomass and diversity indices (average ± standard error).

Family	Specie	Patch		
		NG	LG	SG
Asteraceae	*Achillea millefolium* L.	1.11% ^a^	5.63% ^a^	2.90% ^a^
	*Centaurea nigrescens* Willd.			
	*Cirsium eriophorum* (L.) Scop.			
Apiaceae	sp.	0% ^a^	0% ^a^	1.96% ^a^
Campanulaceae	*Campanula rotundifolia* L.	0% ^a^	0.03% ^a^	0% ^a^
Caryophyllaceae	*Stellaria graminea* L.	0.17% ^a^	0.14% ^a^	0% ^a^
Clusiaceae	*Hypericum perforatum* L.	5.47% ^a^	0.20% ^a^	0% ^a^
Fabaceae	*Trifolium pratense* L.	0% ^a^	0.68% ^a^	0.36% ^a^
	*Trifolium repens* L.			
Poaceae	*Deschampsia caespitosa* (L.) Beauv.	**67.64% ^b^**	**79.50% ^a^**	**72.00% ^a^**
	*Festuca sp.*			
	*Phleum alpinum* L.			
	*Poa alpina* L.			
Polygonaceae	*Rumex acetosa* L.	**18.47% ^a^**	**1% ^b^**	**6.71% ^ab^**
	*Rumex alpinum* L.			
Primulaceae	*Primula elatior* (L.) Hill.	0%	0.18%	0%
Ranunculaceae	*Ranunculus acris* L.	**0% ^b^**	**3.86% ^ab^**	**7.17% ^a^**
Rosaceae	*Alchemilla vulgaris* L.	1.54% ^a^	7.93% ^a^	5.98% ^a^
Urticacae	*Urtica dioica* L.	5.02% ^a^	0% ^a^	2.47% ^a^
other		0.58% ^a^	0.84% ^a^	0.05%
Number of species m^−2^		**8.00 ± 0.71 ^b^**	**11.80 ± 1.39 ^a^**	**8.40 ± 0.40 ^b^**
Indices of Shannon		1.24 ± 0.05 ^a^	1.17 ± 0.13 ^a^	1.15 ± 0.08 ^a^
Indices of Eveness		0.64 ± 0.03 ^a^	0.49 ± 0.08 ^a^	0.55 ± 0.04 ^a^

Letters show the significance of ANOVA at *p* < 0.05, the significant differences between management regimes are marked in bold.

**Table 2:** Concentration of N, C/N ratio, isotopic composition of N (δ^15^N_veg_) and C (δ^13^C_veg_) of green tissues, aboveground biomass and N stock in 5 selected plant species (average ± standard error).

Species	Patch	N,	C/N Ratio	δ^13^C_veg_	δ^15^N_veg_	Biomass	N Stock
		%		‰	‰	g m^−2^	kg ha^−1^
*Deschampsia caespitosa* (L.) Beauv.	NG	**1.** **8** ** ± 0.** **1 ^b^**	**23.** **8 ± ** **1.** **7** **^a^**	−27.8 ± 0.4 ^a^	1.4 ± 0.2 ^a^	**45.0 ± 15.1 ^b^**	**6.1 ± 0.9 ^b^**
	LG	**2.** **3** ** ± 0.** **2 ^ab^**	**18.** **9 ± 2.0 ^ab^**	−26.9 ±0.3 ^a^	1.6 ± 0.9 ^a^	**108.2 ± 23.0 ^a^**	**26.5 ± 7.6 ^a^**
	SG	**2.** **5** ** ± 0.** **1 ^a^**	**17.0** ** ± 1.** **1 ^b^**	−26.9 ± 0.2 ^a^	2.0 ± 0.4 ^a^	**91.3 ± 17.9 ^a^**	**22.3 ± 3.5 ^a^**
*Festuca sp.*	NG	**1.** **9** ** ± 0.** **1 ^b^**	**22.** **3** ** ± 0.** **6** **^a^**	−27.8 ± 0.2 ^a^	1.4 ± 0.3 ^a^	**94.0 ± 32.1 ^a^**	**17.8 ± 6.3 ^a^**
	LG	**2.** **3** ** ± 0.** **2 ^ab^**	**19.** **7** ** ± 1.** **7 ^ab^**	−28.0 ± 0.3 ^a^	0.9 ± 0.7 ^a^	**10.9 ± 8.2 ^b^**	**2.5 ± 1.8 ^b^**
	SG	**3.** **1** ** ± 0.** **3 ^a^**	**14.0** ** ± 1.** **1 ^b^**	−28.0 ± 0.2 ^a^	2.3 ± 0.5 ^a^	**7.1 ± 1.9 ^b^**	**2.2 ± 1.9 ^b^**
*Achillea millefolium* L.	NG	2.2 ± 0.3 ^a^	20.8 ± 2.7 ^a^	**−30.** **3** ** ± 0.** **1 ^a^**	0.7 ± 0.5 ^a^	3.1 ± 2.1 ^a^	0.5 ± 0.3
	LG	2.9 ± 0.3 ^a^	14.8 ± 1.7 ^a^	**−29.** **6** ** ± 0.** **1 ^b^**	1.6 ± 0.7 ^a^	4.7 ± 1.3 ^a^	1.3 ± 0.2
	SG	2.7 ± 0.3 ^a^	16.1 ± 1.1 ^a^	**−29.** **7** ** ± 0.** **1 ^b^**	1.6 ± 0.4 ^a^	4.6 ± 1.7 ^a^	0.8 ± 0.1
*Alchemilla vulgaris* L.	NG	**2.** **5** ** ± 0.** **2 ^b^**	**17.** **4** ** ± 1.** **1 ^a^**	**−28.** **6** ** ± 0.** **1** **^a^**	1.1 ± 0.1 ^a^	**4.3 ± 1.3 ^c^**	**1.1 ± 0.4 ^c^**
	LG	**2.** **7** ** ± 0.** **2 ^b^**	**15.** **6** ** ± 1.** **3 ^ab^**	**−27.** **3** ** ± 0.** **2 ^b^**	0.8 ± 0.6 ^a^	**13.3 ± 3.7 ^b^**	**3.4 ± 0.8 ^b^**
	SG	**3.** **9** ** ± 0.** **1 ^a^**	**11.** **5** ** ± 0.** **2 ^b^**	**−26.** **7** ** ± 0.** **1 ^c^**	−1.7 ± 0.3 ^a^	**20.4 ± 2.6 ^a^**	**7.** **5** ** ± 0.8 ^a^**
*Rumex acetosa* L.	NG	3.9 ± 0.4 ^a^	11.8 ± 1.5 ^a^	−27.5 ± 0.1 ^a^	1.9 ± 0.4 ^a^	**39** **.8 ±** **13.5 ^a^**	**13.5 ± 0.2 ^a^**
	LG	2.8 ± 0.2 ^a^	14.6 ± 0.9 ^a^	−28.4 ± 0.8 ^a^	3.4 ± 1.4 ^a^	**1.** **9** ** ± 0.** **6 ^b^**	**0.6 ± 0.3 ^b^**
	SG	3.3 ± 0.5 ^a^	13.4 ± 2.3 ^a^	−27.9 ± 0.2 ^a^	4.4 ± 1.3 ^a^	**8.2 ± 3.2 ^b^**	**3** **.1 ±** **1** **.3 ^b^**

Letters show the significance of ANOVA at *p* < 0.05, the significant differences between management regimes are marked in bold.

**Table 3:** Bovine faeces chemical characteristics (average ± standard error): concentration of C and N, C/N ratio and isotopic composition of N (δ^15^N) and C (δ^13^C).

Characteristics	Av. Value
δ^13^C, %	−28.65 ± 0.12
δ^15^N, %	1.91 ± 0.41
N, %	1.89 ± 0.19
C, %	43.68 ± 1.43
C:N	23.42 ± 1.52

## Data Availability

Data set available on request to corresponding author.

## Supplementary Materials

The following supporting information can be downloaded at: https://www.mdpi.com/article/10.3390/plants11162121/s1, Table S1. Concentration of N, C/N ratio and isotopic composition of N (δ15Nveg) and C (δ13Cveg) in roots of 5 selected plant species (average ± standard error); Figure S1: Cumulative respiration after 52 days of incubation of soil in differently managed plots (mean ± standard error); Figure S2: Extracellular enzyme soil activity per unit of SOC (mean ± standard error) (a) and per unit of MBC (mean ± standard error) (b). Cel = β-cellobiohydrolase; Chit = Chitinase; β-Glu = β-glucosidase; α-Glu = α-glucosidase; Pho = acid phosphatise; Aryl = arylsulphatase; Xyl = β-xylosidase; But = butyrate esterase; Leu = leucine-aminopeptidase; Figure S3: Relation of Shannon microbial diversity (Hsoil) and synthetic enzymatic index of C-related enzymes (SEI-C) with vegetation species richness in LG plots; Figure S4: Relation of N concentration of green tissues of D. caespitosa with δ15Ns of soil.
