# Supplementary material for: Consequences of Grazing Cessation for Soil Environment and Vegetation in a Subalpine Grassland Ecosystem
Source: Plants (Basel). 2022 Aug 15;11(16):2121. doi: 10.3390/plants11162121 (PMC9416782; doi:10.3390/plants11162121)
Supplement: Supplementary file 1 [file plants-11-02121-s001.zip › plants-1782689-SI.pdf]

Supplementary material

Table S1. Concentration of N, C/N ratio and isotopic composition of N ($\delta^{15}N_{veg}$) and C ($\delta^{13}C_{veg}$) in roots of 5 selected plant species (average ± standard error).

**Species**	**Patch**	**N, %**	**C/N ratio**	**$\delta^{13}C_{veg}$, ‰**	**$\delta^{15}N_{veg}$, ‰**
Deschampsia caespitosa (L.) Beauv.	NG	0.77 ± 0.12[a]	59.86 ± 10.43[a]	-27.41 ± 0.19[a]	0.67 ± 0.52[a]
	LG	0.90 ± 0.07[a]	50.90 ± 6.19[a]	-27.26 ±0.11[a]	1.04 ± 0.39[a]
	SG	0.91 ± 0.06[a]	50.56 ± 3.15[a]	-27.43 ± 0.23[a]	1.55 ± 0.27[a]
Festuca sp.	NG	1.02 ± 0.20[a]	43.95 ± 7.98[a]	-27.61 ± 0.30[a]	1.15 ± 0.35[a]
	LG	0.88 ± 0.06[a]	47.00 ± 2.08[a]	-28.15 ± 0.17[a]	0.72 ± 0.52[a]
	SG	1.04 ± 0.12[a]	45.751 ± 16.51[a]	-27.78 ± 0.10[a]	1.28 ± 0.41[a]
Achillea millefolium L.	NG	1.51 ± 0.98[a]	29.78 ± 4.27[a]	**-29.78 ± 0.36[a]**	0.51 ± 0.42[a]
	LG	1.31 ± 0.13[a]	34.44 ± 3.48[a]	**-28.63 ± 0.09[b]**	0.78 ± 0.68[a]
	SG	1.43 ± 0.03[a]	28.88 ± 1.33[a]	**-28.62 ± 0.15[b]**	1.32 ± 0.09[a]
Alchemilla vulgaris L.	NG	0.94 ± 0.10[a]	47.09 ± 5.08[a]	**-28.03 ± 0.34[a]**	1.02 ± 0.23[a]
	LG	0.96 ± 0.11[a]	54.70 ± 6.20[ab]	**-27.13 ± 0.12[b]**	0.63 ± 0.67[a]
	SG	1.17 ± 0.15[a]	39.80 ± 4.53[b]	**-27.26 ± 0.18[c]**	-0.23 ± 0.45[a]
Rumex acetosa L.	NG	0.84 ± 0.06[a]	51.94 ± 3.68[a]	**-26.61 ± 0.09[a]**	0.11 ± 0.49[a]
	LG	1.07 ± 0.13[a]	41.93 ± 5.23[a]	**-27.71 ± 0.85[ab]**	1.63 ± 1.21[a]
	SG	1.08 ± 0.21[a]	48.10 ± 10.22[a]	**-27.35 ± 0.07[b]**	2.13 ± 1.24[a]

Letters show the significance of ANOVA at $p < 0.05$, the significant differences between management regimes are marked in bold.

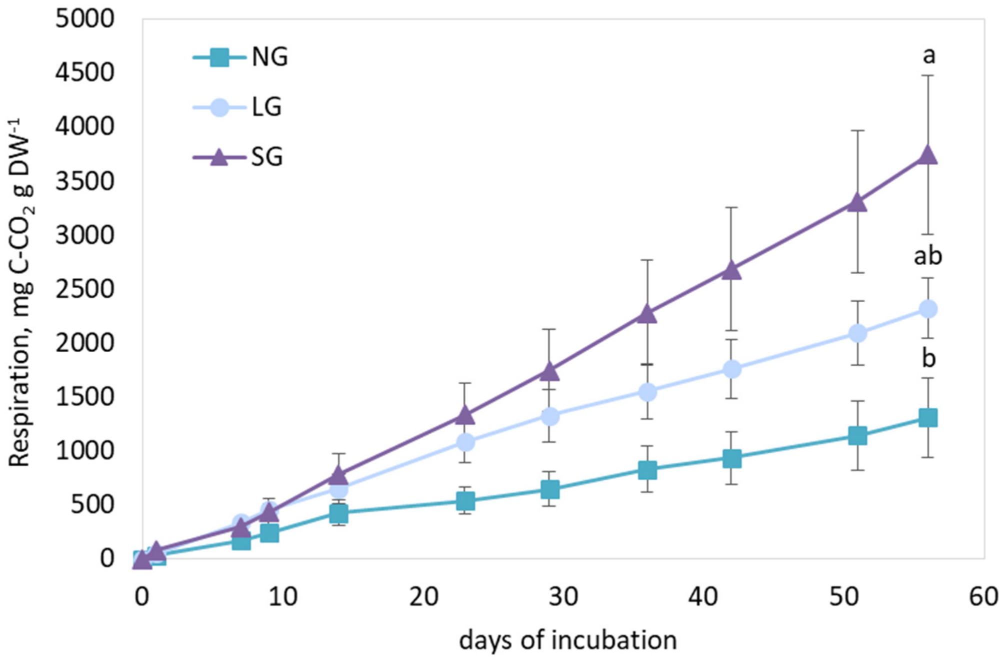

Figure S1. Cumulative respiration after 52 days of incubation of soil in differently managed plots (mean ± standard error). Letters next to the curves show the significance of ANOVA at $p < 0.05$.

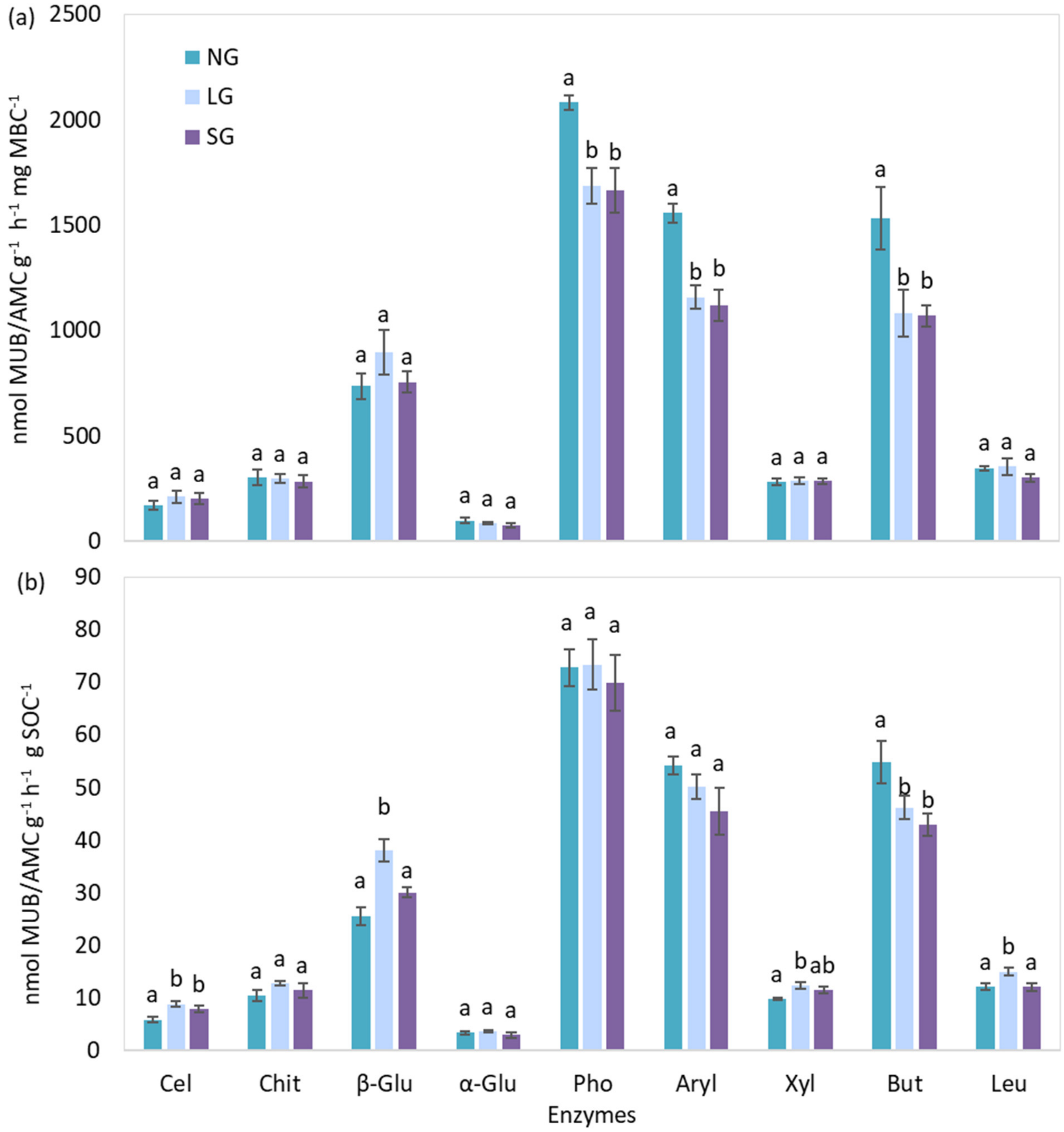

Figure S2. Extracellular enzyme soil activity per unit of SOC (mean ± standard error) (a) and per unit of MBC (mean ± standard error) (b). Cel = β-cellobiohydrolase; Chit = Chitinase; β-Glu = β-glucosidase; α-Glu = α-glucosidase; Pho = acid phosphatise; Aryl = arylsulphatase; Xyl = β-xylosidase; But = butyrate esterase; Leu = leucine-aminopeptidase. Letters next to the bars show the significance of ANOVA at $p < 0.05$.

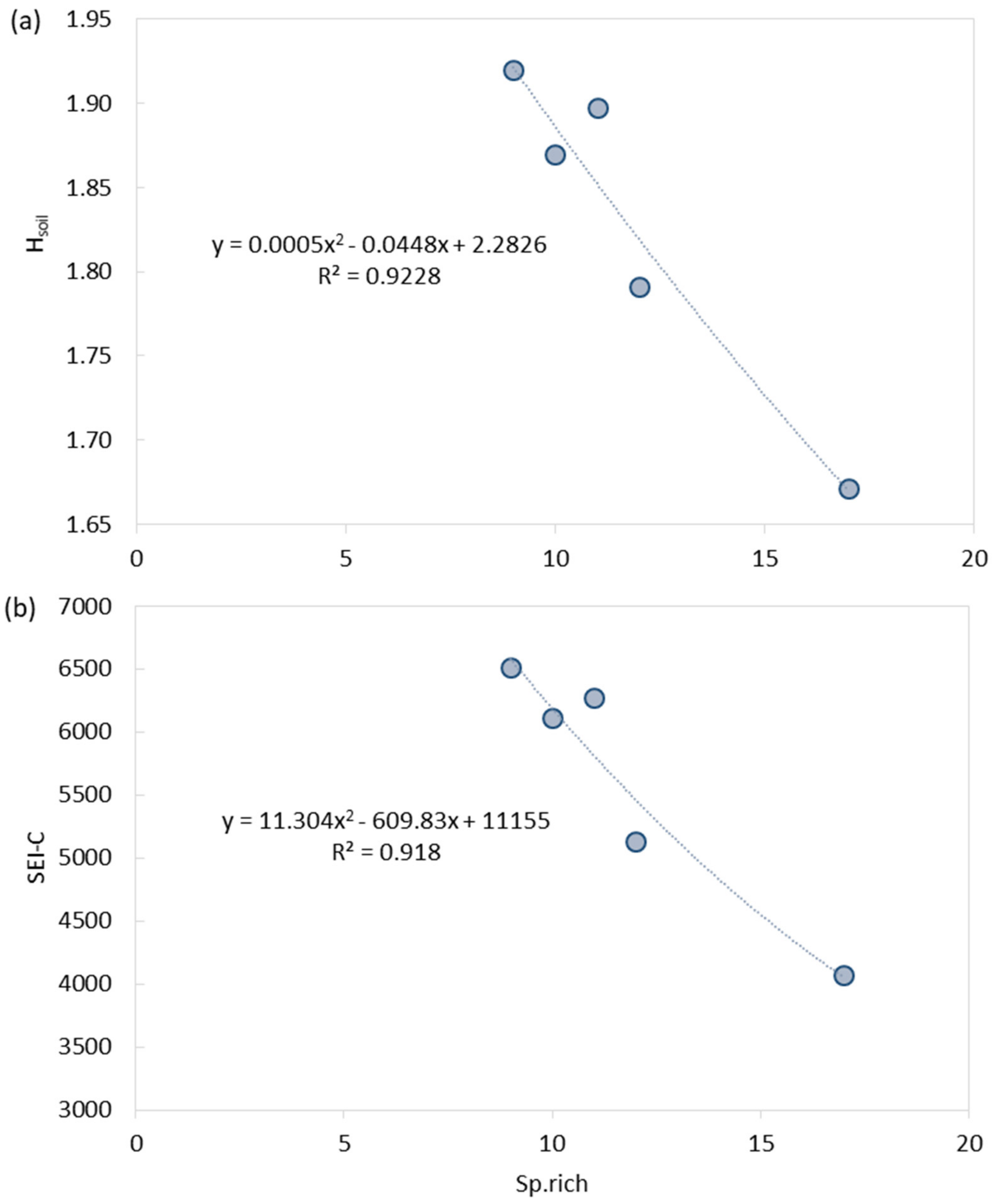

Figure S3. Relation of Shannon microbial diversity (H_{soil}) (a) and synthetic enzymatic index of C-related enzymes (SEI-C) (b) with vegetation species richness (Sp.rich) in LG plots.

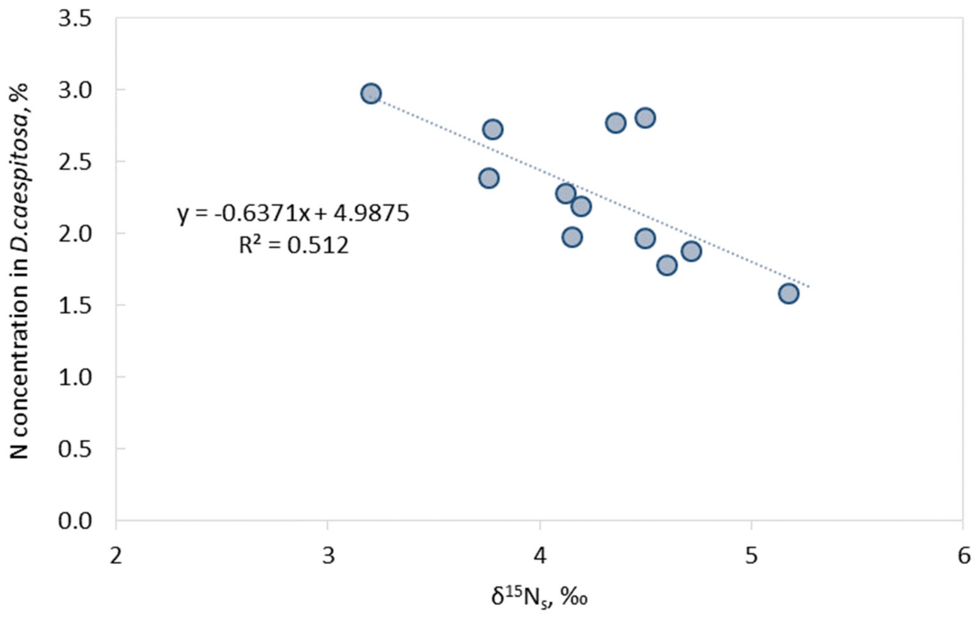

Figure S4. Relation of N concentration of green tissues in *D. caespitosa* with $\delta^{15}N$ of soil ($\delta^{15}N_s$).
